# Resonant and Sensing Performance of Volume Waveguide Structures Based on Polymer Nanomaterials

**DOI:** 10.3390/nano10112114

**Published:** 2020-10-24

**Authors:** Tatiana Smirnova, Volodymyr Fitio, Oksana Sakhno, Pavel Yezhov, Andrii Bendziak, Volodymyr Hryn, Stefano Bellucci

**Affiliations:** 1 Institute of Physics of NASU, Prospect Nauky, 46, 03028 Kyiv, Ukraine; smirnova@iop.kiev.ua (T.S.); pviezhov@gmail.com (P.Y.); volodymyr.o.hryn@gmail.com (V.H.); 2 Institute of Telecommunications, Radioelectronics and Electronic Engineering, Department of Photonics, Lviv Polytechnic National University, Bandera Street, 12, 79013 Lviv, Ukraine; volodymyr.m.fito@lpnu.ua (V.F.); andrii.v.bendziak@lpnu.ua (A.B.); 3 Fraunhofer Institute for Applied Polymer Research, Geiselbergstraße, 69, 14476 Potsdam-Golm, Germany; Oksana.Sakhno@iap.fraunhofer.de; 4 Frascati National Laboratory—National Institute of Nuclear Physics (INFN), Via Enrico Fermi, 54, 00044 Frascati (RM), Italy

**Keywords:** photocurable organic-inorganic nanocomposite, resonant waveguide structures, nanosized particles, holographic biochemical sensors

## Abstract

Organic–inorganic photocurable nanocomposite materials are a topic of intensive research nowadays. The wide variety of materials and flexibility of their characteristics provide more freedom to design optical elements for light and neutron optics and holographic sensors. We propose a new strategy of nanocomposite application for fabricating resonant waveguide structures (RWS), whose working principle is based on optical waveguide resonance. Due to their resonant properties, RWS can be used as active tunable filters, refractive index (RI) sensors, near-field enhancers for spectroscopy, non-linear optics, etc. Our original photocurable organic–inorganic nanocomposite was used as a material for RWS. Unlike known waveguide structures with corrugated surfaces, we investigated the waveguide gratings with the volume modulation of the RI fabricated by a holographic method that enables large-size structures with high homogeneity. In order to produce thin photosensitive waveguide layers for their subsequent holographic structuring, a special compression method was developed. The resonant and sensing properties of new resonant structures were experimentally examined. The volume waveguide gratings demonstrate narrow resonant peaks with a bandwidth less than 0.012 nm. The Q-factor exceeds 50,000. The sensor based on waveguide volume grating provides detection of a minimal RI change of 1 × 10^−4^ RIU. Here we also present the new theoretical model that is used for analysis and design of developed RWS. Based on the proposed model, fairly simple analytical relationships between the parameters characterizing the sensor were obtained.

## 1. Introduction

Organic–inorganic nanocomposites have been intensely investigated in recent decades since they create a basis for the development of components and devices for optics and photonics [1,2,3,4]. These materials usually consist of optically transparent matrices including nano-sized particles (NPs) of different natures, which govern the optical, non-linear optical and plasmonic properties of nanocomposites.

Compared to glass and quartz, polymer matrices possess several advantages. Firstly, it is easier to introduce NPs of various shapes and sizes into a polymer matrix that provides control over the nanocomposite properties. In addition, special technologies for the synthesis of polymers with controlled chemical and physical properties are currently developed that allow creating nanomaterials for particular target applications, for example [5,6].

The photocurable nanocomposites are self-processing materials, which form polymer–NP nanostructures due to the photopolymerization process. NPs can be randomly distributed in a matrix under spatially uniform radiation or they can create the ordered structures, which reproduces the spatial distribution of the polymerizing radiation. The stable ordered structures are formed as a result of diffusion redistribution of the nanocomposite components upon polymerization in a spatially inhomogeneous light field [7,8,9,10,11,12]. The use of photopolymerizable nanocomposites allows the fabrication of one-, two-, and three-dimensional periodic structures of large size and high uniformity by a one-step method of holographic lithography, for example [13,14,15,16] and references therein. The holographic method also provides precise control over symmetry and periodicity of the structures. The performance of photopolymerizable nanocomposites was analyzed in detail in the review [12].

We have developed and studied a series of photocurable nanocomposites based on readily available commercial acrylate monomers, doped with lab-developed and commercial NPs like Au, Ag, TiO_2_, ZrO_2_, SiO_2_, CdS/ZnS, LaPO_4_ [17,18].

Typical modulation of the refractive index (RI), Δn, of all these materials exceeds 0.02. The highest achieved values of Δn were 0.048 for ZrO_2_ NPs [19] and 0.042 in the case of the composite with Ag NPs [18]. Those are close to the maximum possible Δn values for photopolymerizable nanocomposites [12]. To our knowledge, these values are the highest ones reported in academic literature.

A sub-micrometer optical structuring of the nanocomposites significantly expands the field of their possible applications. Periodic structures based on the nanocomposites can be used as holographic diffractive optical elements (DOE) for light control or for displays [12,17], as DOE with ultrahigh spectral dispersion [20,21], and as distributed feedback cavities of waveguide lasers [22,23]. In 2010, the concept of using periodic structures based on the nanocomposites for neutron optics was proposed and successfully implemented [12,24]. Another field for the nanocomposite’s application is the development of holographic biochemical sensors (reviews [25,26,27,28] and references therein). In modern conditions of the growing danger of chemical and biological environmental pollution such sensors can be singled out as one of the most important applications. The principle of the sensors’ operation is based on using of holographic gratings that respond to small changes in their environment. Transmission or reflection gratings which contain sensitive components that can change grating parameters under the influence of the test analytes are usually used. These effects can be detected by the change in the diffraction efficiency of the gratings or by angular or spectral response. Reflection Bragg gratings with high spectral selectivity are particularly sensitive. The change in the Bragg peak spectral position correlates with the concentration of the absorbed analyte that is being measured. Such sensors can be applied for express analyses by checking the color variation with the naked eye. 

In this paper, we will consider a new application of photocurable nanocomposites to develop resonant waveguide structures (RWS), whose working principle is based on waveguide resonance in a dielectric grating placed on a dielectric substrate. The dielectric resonant structures have been a topic of intensive research since the 1960s. Due to the low extinction coefficient of dielectrics, the RWS are characterized by low losses and, accordingly, narrow resonances. As a result, various RWS were implemented for application as active tunable filters, laser mirrors, refractive index (RI) sensors, near-field enhancers for fluorescent and Raman spectroscopy, enhancement of non-linear effects, etc. The development of this research area is comprehensively reflected in the review of Quaranta et al. [29].

The resonance concept is a fundamental basis for the sensor applications. At specific wavelengths and at certain incident angles of white light on the resonant structure coupling of the propagating waves, diffracted by the periodic structure, to the waveguide eigenmodes take place. Under this condition one of the diffracted waves transforms from a traveling wave to an evanescent one, no light is transmitted, and strong peaks appear in the reflection spectrum of the RWS. The central wavelengths of these peaks depend on the grating period, on the RI of the substrate, the grating and the environment. When the analyte under study is placed on a structure surface, the RI of the environment changes and the resonant peak shifts in wavelength. Thus, the RWS can be used as on-chip RI sensors. In reference [30,31], a theory of spectroscopic monitoring method of the RI of media near the structure surface was proposed. It was termed grating light reflection spectroscopy. The experimental implementation of the method is also described in these papers. Later, Cunningham et al. [32,33] proposed the RI and fluorescence sensors, in which 1D- and 2D-resonant structures were used. This concept has been further developed and applied to produce compact and inexpensive commercial biochemical sensors. A wide variety of metal-based plasmonic and dielectric-based photonic RI sensors have been developed to date, and their comparative characteristics, advantages and disadvantages are presented in references [29,34,35]. It should be noted that the sensitivity of the sensors based on resonant structures significantly exceeds the sensitivity of holographic sensors.

The main types of the RWS, which are currently implemented in practice, are the corrugated waveguide structures (the example is a commercial Corning Epic 384-well RI biosensor) [29,34,35] and references therein. The sensitivity of RI sensors is determined by the minimal detectable change in the spectral position of the resonant peak and, accordingly, depends on the accuracy with which the resonant peak spectral position may be found. The spectral FWHM (full width at half maximum) of the resonant peaks for corrugated structures usually exceeds 1 nm. In addition, the resonance peaks can broaden due to the variation in filling factor and groove depth that can occur during the fabrication with the lithographic or ultraviolet (UV)-embossing methods [36,37]. In order to increase the accuracy of the RI definition, geometry and parameters of the structures are varied and different methods of signal processing are used. As a result, a design of all-dielectric RI sensor based on a simple 1D-grating was proposed, which ensures the bandwidth (FWHM) decrease down to 0.02 nm [38]. The sensor properties of the corrugated waveguide structures will be considered in more detail when discussing the experimental results.

The nanocomposite RWS with the volume modulation of dielectric permittivity fabricated by the holographic method are free of “fabrication errors” and, according to our estimates, possess a bandwidth of about 0.002 nm. Their high spatial uniformity and narrow resonances are the prerequisites for using the nanocomposite RWS as RI sensors. 

Here we propose a simple and cheap method for fabrication of photosensitive waveguide layers from the nanocomposite for subsequent holographic structuring. We investigate the resonance properties of nanocomposite RWS and demonstrate the possibility of their use as RI sensors. We also present the new theoretical model for the analysis and design of the RI sensors.

## 2. Materials and Methods 

### 2.1. Materials

**Figure 1:**
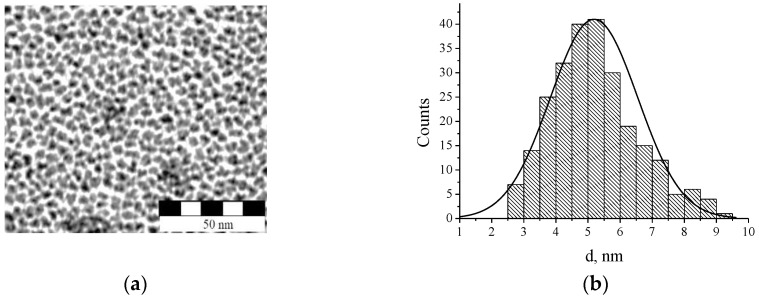
(**a**) Transmission electron microscopy (TEM) image of the grating with the LaPO_4_ nanoparticles (NPs) and (**b**) histogram of the NP size distribution (the area is 3.5 µm × 3.5 µm; the average diameter is 5.24 nm; the standard deviation is 1.37 nm).

The organic-inorganic photosensitive nanocomposite for the fabrication of RWS was prepared using earlier developed [39] and now optimized technology. As an organic matrix of the nanocomposite a blend of two low-polar acrylate monomers, a mono-functional isobutyl acrylate (IBA, n = 1.476) and a multi-functional acrylate monomer Sartomer SR444 (n = 1.481), in the ratio of IBA 75 wt.% and SR444 25 wt.%, was used. Both monomers were purchased from Aldrich and used as received. To this, 1.5 wt.% of photoinitiator Irgacure 1700 (Ciba) was added to the monomer blend to provide the material sensitivity to UV light. The inorganic NPs series X green (LaPO_4_:Ce,Tb) were purchased from Fraunhofer Center for Applied Nanotechnology Fraunhofer Germany (CAN) as a viscous colorless gel. In order to provide high compatibility with low-polar acrylates monomers the surface of the NPs was functionalized with an organic amine-based substance (CAN). After washing and drying of the gel the NP powder was obtained. The transmission electron microscopy (TEM) image of the NPs is presented in Figure 1a (Field-Emission Electron Microscope JEOL JEM-2200 FS). The average nanoparticles size was estimated of 5.24 nm. Inorganic core was found of about 80 wt.% (according to the thermal gravimetric analysis method). 

The required amount of the NPs is firstly dispersed in pentane using an ultrasonic bath. After that, the NP dispersion in solvent was added to the monomer blend and mix with a magnetic stirrer at room temperature. After complete solvent evaporation, the low-viscous optically transparent organic–inorganic UV-sensitive nanocomposite is ready. 

According to the manufacturer’s data, the absorption band of NPs lies in the spectral range of 200–300 nm. This means that the introduction of NPs into the light-sensitive monomer mixture does not affect the absorption of actinic radiation (355 nm) by the initiator. The polymer grating is practically transparent in the spectral range from 400 to 800 nm, which is required for further applications of the resonant structures. 

The developed nanocomposites perform relative high optical transparency even at 32 wt.% loading of the NPs in the monomer blend and of about 2-month shelf-life in a lab. The optimal content of the NPs in polymer matrix was adjusted during holographic fabrication of the gratings (the details are presented in [17,39]). The largest refractive index modulation amplitude ($n_{1}$) of the structures along with the smallest light scattering in the layers of desired thickness was achieved for the NPs content of 28–30 wt.%.

### 2.2. Holographic Fabrication and Characterization of Resonant Waveguide Structures (RWS)

Holographic recording of the RWSs was carried out using a symmetric two-beam setup for the fabrication of transmission gratings. The beam from the UV laser (Genesis SLM, Coherent), operating at 355 nm (*λ_rec_*) with maximum output power of 110 mW, was firstly split in two beams possessing *s*-polarization and equal intensity and then overlapped on the sample position forming an interference pattern. According to the above calculations of the RWS parameters the angle between the interfering beams was adjusted of 52.8° that provides the creation of volume gratings with the spatial period Λ = 399 nm. The photosensitive samples in the form of the glass cells, with a thin nanocomposite layer in between, were exposed for 150–180 s at the intensity between of 15–25 mW/cm^2^. In order to completed the polymerization around the grating spot, the sample were exposed to low UV irradiation using a Philips UV Lamp for 5 min. The nanocomposite gratings are formed directly upon holographic exposure due the lateral light-induced diffusion redistribution of the nanocomposite components. It provides a stable regular periodicity of inorganic NPs in the polymer film that leads to volume modulation of the grating RI (more details of the fabrication of the nanocomposite gratings see are, for example, in [17,18,39]. The grating formation was monitored in real time by the diffraction of the *s*-polarized He-Ne laser beam of *λ_t_* = 633 nm, placed at a corresponding Bragg angle. The fabricated gratings perform the Bragg properties: the values of the Cook–Klein parameters were estimated as 16 and 36 for the grating thicknesses 1 and 2 µm, respectively. The diffraction signals were measured with two silicon detectors and the data were acquired with a computer. The diffraction efficiency of the grating (*η*) was estimated as the intensity of the −1^st^ order beam (I_−1_) divided by the sum of I_−1_ and I_0_ (intensity of the 0^th^ order beam) as *η*(t) = I_−1_(t)/(I_−1_(t) + I_0_(t)) in order to exclude the Fresnel reflection of the substrates, scattered light and linear absorption of the nanocomposite layers. The amplitude of the refractive index modulation, $n_{1}$, ($n_{1}$ = Δn/2), of the fabricated RWS was calculated using Kogelnik’s formula [40]:

(1) $$n_{1}=\left(\frac{\lambda_{t}cos\theta_{B}}{\pi d}\right)asin\left(\sqrt{\eta }\right)$$

where θ*_B_* is the Bragg angle within the medium, *d* is the thickness of the grating. 

The thickness of polymer films and gratings were examined using a Dektak Veeco 150 profilometer and a Linnik micro-interferometer with a charge-coupled device (CCD). The film thickness was estimated as a height of the step at the polymer film/substrate interface. The profilometer measures the step height up to 0.1 nm. The interferometric method allows measuring the displacement of the interference pattern by 0.01 nm. In the case of the spin-coated layers, when a polymer film was covered over the entire substrate, some part of the film was mechanically scratched away to expose the substrate. The height was measured on both sides of the scratch. The height (film thickness) was typically measured in four or six places of the film without grating or around the grating area. The results of both methods were almost compatible. The spread of the obtained values did not exceed 1.5% for gratings and of about 3% for the spin-coated films. The averaged thickness value was used for the calculation of *n*_1_ and for the theoretical estimations of the RWSs resonant characteristics. 

The refractive indices of the monomers/polymers, the nanocomposites and the fluids under study were measured by Abbe refractometer.

### 2.3. Reflection Spectra Measurements

For the measurement of the reflection spectrum, the grating-waveguide was mounted on a dual axis rotation stage with the azimuth/polar angular resolution of 20″/0.2°. This allowed us to measure precisely the incident angle θ of the probing radiation and to align the grating fringes parallel to the *Z*-axis of the laboratory coordinate system (normal to the surface of the optical setup).

**Figure 2:**
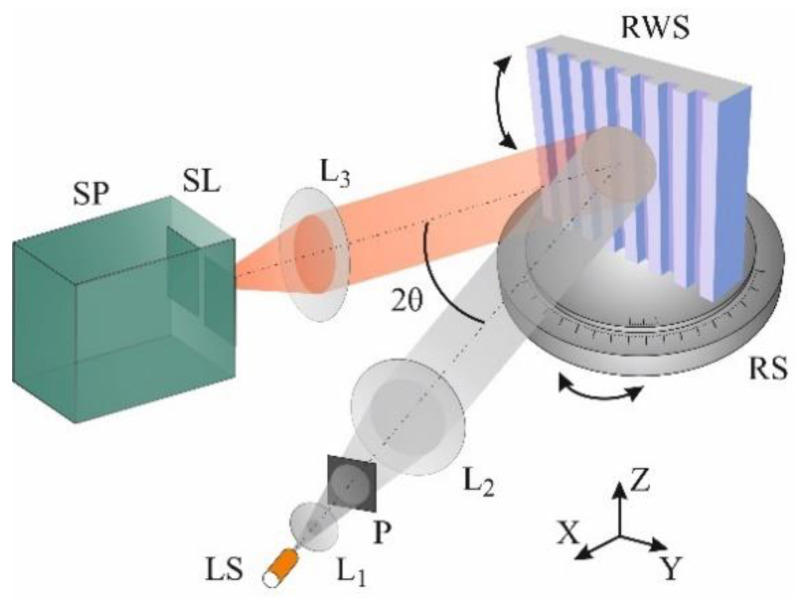
Schematic of reflection experiment setup: LS is the light source, L_1_, L_2_ and L_3_ are the lenses, P is the polarizer, RS is the rotation stage, RWS is the resonant waveguide structure on the rotation stage RS, SP is the spectrometer with a slit SL, 2θ is the angle between the beams.

A white light-emitting diode (LED) with a maximum radiation near 585 nm was used as a light source (Figure 2). A plane wave from the light source formed by a collimator comprising an achromatic lens was directed to the sample at an angle θ, in such a way that azimuthal angle between incident and reflected beams equals to 2θ. The polarization state of the light wave, TE or TM (field vector, **E**, is parallel or perpendicular to the *Z*-axis) could be selected with help of polarizer. In order to suppress the Fresnel reflection at the substrate/air interface a neutral gray filter was fixed on the outer side of the grating’s substrate using an immersion liquid.

The reflected light was collected to a charge-coupled device—spectrometer ACTON SpectraPro 2500i, equipped with a 2400 lines/mm grating and EEV 256 × 1024 OE CCD30 detector with 16 bit dynamic range. The recorded wavelength range was 12 nm; a single pixel of CCD corresponded to 0.012 nm. To obtain the peaks with sufficient accuracy the reflection spectra were recorded with the accumulation over thirty measurements

## 3. Results

**Figure 3:**
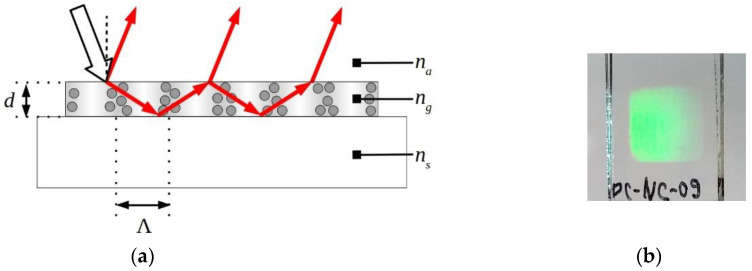
(**a**) RWS structure: *d* is the layer thickness; Λ is the structure period; *n_a_*, *n_g_*, and *n_s_* are the refractive indices of the analyte, the grating, and the substrate, respectively; (**b**) the image of a holographic RWS fabricated in the organic-inorganic nanocomposite.

The RWS based on the organic–inorganic nanocomposite is schematically shown in Figure 3a. A planar polymer waveguide with periodically arranged NPs is located on an optically transparent substrate. The Figure 3b demonstrates the diffraction pattern of white light on a grating-waveguide.

The volume spatial modulation of the NP concentration creates the spatial modulation of the waveguide RI. This structure possesses the resonant phenomena termed a guided-mode resonance. 

As stated above, the resonance conditions manifest themselves by appearance of strong peaks in the reflection (transmittance) spectrum of the RWS. The variation in $n_{a}$ causes the change in spectral (angular) position of central wavelengths of these peaks. The principle of operation of the RI-sensor is based on measuring the dependence of the spectral (angular) shift of the resonance peak on the change in RI.

### 3.1. Theoretical Analysis of Resonant Properties of RWS

An important step for design and manufacturing of the RWS for sensing applications is the theoretical modeling of this structure, in order to determine the waveguide thickness and the grating period, which ensure the resonance conditions in the selected wavelength range. The parameters of such structures are usually determined by the numerical methods, among which the most common is rigorous coupled-wave (RCWA). In this section, we present a theoretical model developed by the authors for the analysis and design of the proposed RWS.

We studied the waveguide gratings with volume modulation of the RI, Δ$n_{g}$, which does not exceed 0.025. The value of Δ$n_{g}$ is much smaller than the grating average RI, $n_{g}$ > 1.5. Under the circumstances the waveguide mode is perturbed weakly by the grating; and many parameters of such a structure can be determined using only the dependence of the propagation constants of localized waveguide modes on the wavelength and the RI of the studied medium. The same situation holds for the waveguide with a relief grating, the thickness of which is significantly smaller than the waveguide thickness and the wavelength [30].

To describe the resonant properties of the waveguide gratings with extremely low modulation a theoretical model based on RCWA and on classical slab waveguide theory was proposed in reference [41]. This model allowed obtaining analytical expressions to describe the main features of the guided-mode resonance and predicting the ranges of the incident angles and wavelengths within which the resonances can be excited. However, the determination of the resonance wavelengths for different angles of incidence and the structure parameters requires complex calculations using transcendental equations and the coupled wave method. The calculations become significantly more complicated as the number of the waveguide layers increases. In addition, the model does not allow obtaining analytical expressions describing the sensitivity and selectivity of sensors based on RWSs. The propagation constants, β, cannot be found in analytical expressions depending on the waveguide parameters and the wavelength, since they are the solutions of transcendental equation [42].

Therefore, we propose a simpler theoretic model also based on the slab waveguide theory. In this model the original simple numerical method for analysis of waveguide modes in planar gradient waveguides, developed by the authors, is used to analyze the resonant properties of such a structure. This method allows evaluating the propagation constants and the corresponding fields with a high precision accuracy even for multilayer waveguides and obtaining fairly simple analytical expressions for calculating sensor sensitivity [43,44]. The proposed model has a twofold advantage. On the one hand, it simplifies the calculation procedure of the resonance wavelengths for different angles of incidence and structure characteristics. On the other, it allows obtaining the analytical relationship between the sensor sensitivity and parameters of a planar waveguide.

#### 3.1.1. Development of the Approximate Theoretical Model

The proposed approach can be used for the waves with both TE and TM polarization. It was shown in [41] that the resonance peaks for the TM-polarized waves are two orders narrower than for the TE-polarized waves. Since it is difficult in practice to implement a sensor on the base of structure with very narrow resonance peaks (10^−4^–10^−5^ nm), all our studies were carried out for TE-polarized waves.

To achieve the propagation of a waveguide mode with a discrete propagation constant β in the structure shown in Figure 1a, the average RI of the grating ($n_{g}$) must be higher than the RI of the substrate ($n_{s}$) and the analyzed medium ($n_{a}$), $n_{g}$ > $n_{s}$, $n_{a}$. In this case β, the wavelength λ and the RI of the waveguide layers should satisfy the following condition [42]:

(2) $$\frac{2\pi \max\left(n_{a},n_{s}\right)}{\lambda }<\beta <\frac{2\pi n_{g}}{\lambda },$$

If a plane wave with a wavelength *λ*_0_ falls normally on a dielectric grating with a period Ʌ, the following condition must be fulfilled at the waveguide resonance:

(3) $$\beta \left(\lambda_{0}\right)≅\frac{2\pi }{\Lambda },$$

where $\beta \left(\lambda_{0}\right)$ is the propagation constant of a waveguide mode for a given wavelength, which also depends on the RI of the waveguide layers. Note, that reflection coefficient from the grating is equal to one under the resonant conditions. Knowing the wavelength $\lambda_{0}$ and other parameters of the waveguide we can determine the grating period Λ by Equation (3). According to [45] it is possible to solve the inverse problem numerically, i.e., for a given grating period from Equation (3), to define the propagation constant β and, accordingly, the set of wavelengths that have this propagation constant.

If $n_{g}$ = 1.525, $n_{s}$ = 1.515, $n_{a}$ = 1.500, Λ = 399 nm, the waveguide resonance occurs at the wavelength 605.3418 nm for the mode with a propagation constant β = 15.72504 μm^−1^. In this case, Equation (3) is true with the following accuracy:

(4) $$\left(\beta -\frac{2\pi }{\Lambda }\right)/\beta <5\times 10^{-5}.$$

If a plane wave illuminates a grating at the angle θ, the waveguide resonance condition can be written in a slightly different form:

(5) $$\frac{2\pi }{\lambda }\sin\theta \pm \frac{2\pi }{\Lambda }≅\pm \beta \left(\lambda ,n_{a}\right),$$

where θ is the angle of a beam propagation in air. In this case ($n_{g}$ = 1.525, $n_{s}$ = 1.380, $n_{a}$ = 1.500, Λ = 399 nm) at incidence angle 20° and at resonant wavelength 0.7402315 μm^−1^ the following relation is true:

(6) $$\left(\frac{2\pi }{\lambda }\sin\theta -\frac{2\pi }{\Lambda }+\beta \right)/\beta <5\times 10^{-5}.$$

The right and left parts of Equations (3) and (5) are very close to each other due to the small amplitude of the grating RI change. The corresponding difference will tend to zero when the amplitude decreases [41]. Therefore, we can calculate the spectral and angular sensitivities of a sensor if the dependency of the propagation constant of the planar waveguide on the wavelength and the RI of the studied medium is known.

Once the resonant wavelength $\lambda_{0}$ at normal incidence is already determined, we can calculate the approximate values of the resonant wavelengths at an arbitrary incident angle, θ. Expanding the right part of Equation (5) in a neighborhood of $\lambda_{0}$ into a Taylor series and taking first three terms of this expansion, one can obtain from Equations (3) and (5):

(7) $$\frac{2\pi }{\left(\lambda_{0}+∆\lambda \right)}sin\theta =\pm \left[\frac{d\beta }{d\lambda }∆\lambda +\frac{1}{2}\frac{d^{2}\beta }{d\lambda^{2}}{\left(∆\lambda \right)}^{2}\right]$$

The analysis of Equation (7) leads to the conclusion that at an arbitrary incident angle two resonant peaks arise at the wavelength $\lambda_{1}$, smaller than $\lambda_{0}$, and at the $\lambda_{2}$, higher than $\lambda_{0}$. In fact, Equation (7) splits into two equations:

(8) $$F_{1}\left(\Delta \lambda_{1}\right)=\frac{2\pi }{\left(\lambda_{0}+\Delta \lambda_{1}\right)}sin\theta -\left[\frac{d\beta }{d\lambda }\Delta \lambda_{1}+\frac{1}{2}\frac{d^{2}\beta }{d\lambda^{2}}{\left(\Delta \lambda_{1}\right)}^{2}\right]=0,$$

(9) $$F_{2}\left(\Delta \lambda_{2}\right)=\frac{2\pi }{\left(\lambda_{0}+\Delta \lambda_{2}\right)}sin\theta +\left[\frac{d\beta }{d\lambda }\Delta \lambda_{2}+\frac{1}{2}\frac{d^{2}\beta }{d\lambda^{2}}{\left(\Delta \lambda_{2}\right)}^{2}\right]=0.$$

Equations (8) and (9) are easy to solve graphically and the first and second derivatives can be found numerically from the solution of the waveguide equation by the method described in reference [43].

Let us consider the procedure for determining the resonance wavelengths using a model resonant structure with the following parameters: $n_{s}$ = 1.515, $n_{a}$ = 1, $n_{g}$ = 1.525, amplitude of the refraction index change in the grating medium $n_{1}$ = 0.017, grating thickness *d* = 1.3 μm, Λ = 0.399 μm.

**Figure 4:**
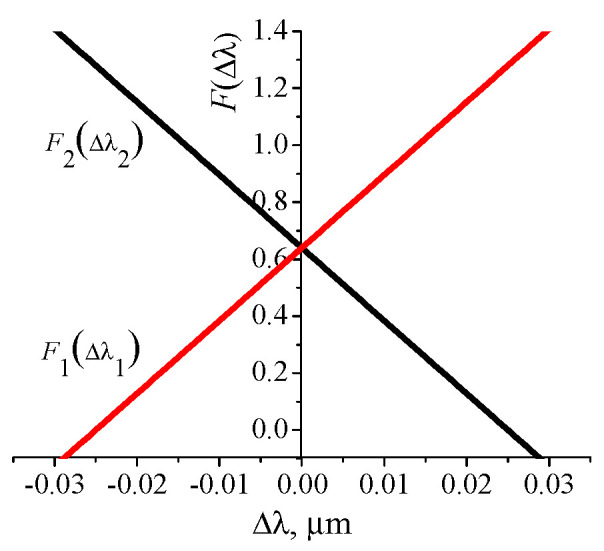
Dependencies $F_{1}\left(∆\lambda_{1}\right)$ and $F_{2}\left(∆\lambda_{2}\right)$ for the angle of incidence on the grating 3.5°.

The resonant wavelength at the normal incidence (*λ*_0_) calculated as indicate above is 0.6053418 μm for these parameters. Then the first *d*β/*dλ* and the second *d*^2^β/*dλ*^2^ derivatives are equal to 26.158647765 μm^−2^ and 86.637 μm^−3^, respectively. Using the obtained values for the graphical solution of Equations (8) and (9), it is possible to determine the shifts of the resonant wavelengths ($∆\lambda_{1},\;∆\lambda_{2}$) at the selected angle of incidence. The dependencies $F_{1}\left(\Delta \lambda_{1}\right)$ and $F_{2}\left(\Delta \lambda_{2}\right)$ obtained at θ = 3.5° are shown in Figure 4, where the roots of Equations (8) and (9) are determined by the intersection of the curves with the abscissa. 

Thus, the predicted resonance wavelengths are equal $\lambda_{1}=\lambda_{0}+∆\lambda_{1}=$ 581.0815 nm, $\lambda_{2}=\lambda_{0}+∆\lambda_{2}=$ 629.6055 nm. The wavelengths calculated with RCWA for an infinite grating, at which the reflection coefficient tends to unity, are 581.138 nm and 629.689 nm, respectively. It can be seen that the predicted wavelengths are very close to the wavelengths determined from the spectral dependencies of the reflection coefficient calculated by the RCWA method when the incident angle is deviated from normal. The proposed method was used for predicting resonant wavelengths in numerical and real experiments to limit the range of λ variation in accurate RCWA calculations. This made it possible to accelerate the procedure for calculating the resonance wavelengths with a change in the incident angle of the light beam on the grating.

Using Equation (5), the resonant wavelengths for various $n_{a}$ can be also determined. Obviously, in this case the propagation constant β depends on λ and $n_{a}$. Thus, when changing $n_{a}$ by $\delta n_{a}$, the resonant wavelength changes from λ to λ+δλ at constant incidence angle of the beam. This leads to the following equation:

(10) $$\frac{2\pi }{\left(\lambda +\delta \lambda \right)}sin\theta \pm \frac{2\pi }{\Lambda }=\pm \beta \left(\lambda +\delta \lambda ,n_{a}+\delta n_{a}\right).$$

Using the procedure described above we obtain:

(11) $$\frac{-2\pi sin\theta }{\lambda^{2}}\delta \lambda =+\left(\frac{\partial \beta }{\partial \lambda }\delta \lambda +\frac{\partial \beta }{\partial n_{a}}\delta n_{a}\right),$$

(12) $$\frac{-2\pi sin\theta }{\lambda^{2}}\delta \lambda =-\left(\frac{\partial \beta }{\partial \lambda }\delta \lambda +\frac{\partial \beta }{\partial n_{a}}\delta n_{a}\right).$$

Let us introduce the following notation: $S_{n}=\frac{\partial \beta }{\partial n_{a}},\;S_{\lambda }=\frac{\partial \beta }{\partial \lambda }$, which can be found using numerical methods, for example, $S_{n}=\frac{\beta \left(\lambda ,n_{a}+\delta n_{a}\right)-\beta \left(\lambda ,n_{a}-\delta n_{a}\right)}{2\delta n_{a}}$. The sensitivity (S), as the ratio of the change in resonant wavelength to the change in $n_{a}$, can be determined from Equation (11) in the following way:

(13) $$S=\frac{\delta \lambda }{\delta n_{a}}=\frac{-S_{n}}{\frac{2\pi sin\theta }{\lambda^{2}}+S_{\lambda }},$$

From Equation (12) we have

(14) $$S=\frac{\delta \lambda }{\delta n_{a}}=\frac{S_{n}}{\frac{2\pi sin\theta }{\lambda^{2}}-S_{\lambda }}.$$

The propagation constant can be expressed as $\beta \left(n_{a},\lambda \right)=\frac{2\pi n_{ef}}{\lambda }$, moreover the relation $max\left(n_{a},n_{s}\right)<n_{ef}<n_{g}$ is true. If $max\left(n_{a},n_{s}\right)$ and $n_{g}$ are close enough, $n_{ef}$ slightly depends on the wavelength, and as a result we obtain:

(15) $$S_{\lambda }=\frac{d\beta }{d\lambda }≅-\frac{2\pi n_{ef}}{\lambda^{2}}=\frac{-\beta }{\lambda }.$$

Taking into account Equation (5), Equations (13) and (14) can be rewritten as:

(16) $$S=\frac{-S_{n}}{\frac{2\pi sin\theta }{\lambda^{2}}-\frac{\beta }{\lambda }}=\frac{\lambda \Lambda }{2\pi }S_{n},$$

(17) $$S=\frac{S_{n}}{\frac{2\pi sin\theta }{\lambda^{2}}+\frac{\beta }{\lambda }}=\frac{\lambda \Lambda }{2\pi }S_{n}.$$

It should also be noted that the sensitivity at the sign “–” in Equation (16) will be greater than the sensitivity at the sign “+” in Equation (17).

The angular sensitivity of the resonance structure can be determined by the same method. Let the wavelength of the probing radiation remains constant. As $n_{a}$ changes the resonance conditions will be satisfied for different θ. Then Equation (5) can be rewritten in the form:

(18) $$\frac{2\pi }{\lambda }sin\theta \pm \frac{2\pi }{\Lambda }≅\pm \beta \left(n_{a}\right).$$

Using the expansion of the right and left parts of the Equation (18) into the Taylor series, the change of the angle of the waveguide resonance because of the change of the $n_{a}$ can be expressed as:

(19) $$\delta \theta =\frac{\pm \frac{d\beta }{dn_{a}}}{\frac{2\pi }{\lambda }cos\theta }\delta n_{a}=\left(\pm \frac{\lambda S_{n}}{2\pi cos\theta }\right)\delta n_{a}=S_{\theta }\delta n_{a},$$

where $S_{\theta }=\frac{\lambda S_{n}}{2\pi cos\theta }$.

The interrelation between the spectral bandwidth, $\delta \lambda_{0.5}$, and the angular bandwidth, $\delta \theta_{0.5},$ can be determined from Equation (5). Differentiating the right and left sides of Equation (5) by the angle θ and the wavelength λ with a constant $n_{a}$, we obtain:

(20) $$\frac{-2\pi sin\theta }{\lambda^{2}}\delta \lambda +\frac{2\pi }{\lambda }cos\theta \delta \theta \pm \frac{d\beta }{d\lambda }\delta \lambda =0$$

and, correspondingly,

(21) $$\delta \theta_{0.5}=\left(\frac{tan\theta }{\lambda }\pm \frac{\lambda S_{\lambda }}{2\pi cos\theta }\right)\delta \lambda_{0.5}.$$

Taking into account Equation (18) and $S_{\lambda }=\frac{d\beta }{d\lambda }≅-\frac{\beta }{\lambda }$, we can rewrite Equation (21) as:

(22) $$\delta \lambda_{0.5}=\Lambda cos\theta \delta \theta_{0.5}$$

Dividing the right part of Equation (16) by the right part of Equation (22) and $S_{\theta }$ by $\delta \theta_{0.5}$, we obtain an identity, meaning that the following fundamental expression is true:

(23) $$\frac{S}{\delta \lambda_{0.5}}=\frac{S_{\theta }}{\delta \theta_{0.5}}=\frac{\lambda S_{n}}{2\pi cos\theta \delta \theta_{0.5}}.$$

Thus, on the basis of the relations obtained, it can be concluded that the sensitivity of the resonance structure increases with the increase in the incidence angle and wavelength. The sensing ability of RI sensors usually can be assessed by spectral sensitivity to RI change (S, in our case) and the figure of merit, FoM (FoM = $S/\delta \lambda_{0.5}$).

#### 3.1.2. Results of the Numerical Modeling of the Waveguide Resonance

Analyzing the dependence of the propagation constants on *λ* and $n_{a}$ we can obtain simple expressions for the spectral and angular sensitivity, that were described in the previous section. However, to determine the spectral or angular FWHM of the resonant peaks, we must study the diffraction of the light beam by the grating which can be done by numerical methods. The numerical waveguide resonance analysis was performed with the RCWA.

**Figure 5:**
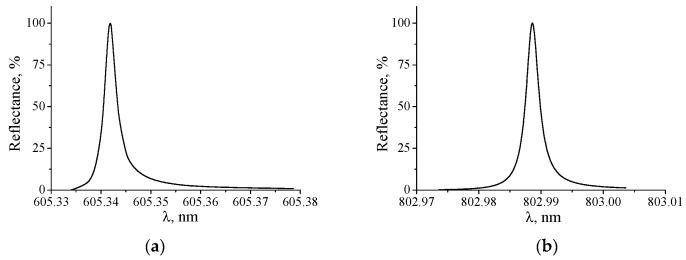
(**a**) Spectral dependence of the reflectance at normal beam incidence on the grating. The $\delta \lambda_{0.5}$ of the spectrum is 0.003 nm; (**b**) spectral dependence of the reflectance at the angle of incidence of 30°. The $\delta \lambda_{0.5}$ of the spectrum is 0.0027 nm.

The most important characteristic of the resonance structures is the spectral FWHM of the resonance peak, which determines the minimal change in the RI, which can be detected by a sensor. In Figure 5a,b the reflection spectra from the structures with the above mentioned parameters are shown. As can be seen from the figures, the $\delta \lambda_{0.5}$ is very small and practically does not change with increasing the incident angle up to 30°. The bandwidth of the angular dependence of the reflection coefficient, calculated for the same parameters, is 0.000417°.

The calculations also show that the change of the polarization state to TM leads to a small shift in the resonance wavelength and to decrease in the bandwidth of the resonance peak by two orders of magnitude. Thus, for the structure with the same parameters at θ = 0°, *λ_res_* = 605.486 nm and $\delta \lambda_{0.5}$ = 3.5 × 10^−5^ nm.

Table 1 shows the resonant wavelengths (long-wave resonance), sensitivities $S_{n}$, $S_{\lambda }$, S, $S_{\theta }$ and FoM depending on $n_{a}$ at θ = 30°. The calculations were carried out using Equations (16) and (19) for the aforementioned parameters of the resonant structure. The resonant wavelengths were determined by the RCWA method. To estimate FoM, we used the value of $\delta \lambda_{0.5}\approx$ 0.003 nm. 

As follows from the Table 1, the ratios calculated for n = 1.500 are approximately equal to: $S/{\delta \lambda }_{0.5}=20.2/0.0025\approx 3.368/0.000417=S_{\theta }/{\delta \theta }_{0.5}$, which is very consistent with Equation (23).

Analyzing Table 1, one can conclude that the sensitivities increase rapidly with increasing $n_{a}$, especially when $n_{a}$ approaches $n_{g}$. The resonant wavelengths vary slightly with a variation of $n_{a}$ from 1 to 1.5. In addition, the sensitivity increases at least three times in the presence of a buffer layer with a low refractive index on the substrate under the grating, comparing to the sensitivity value when it is absent.

We point out that all estimations were carried out for a single-mode waveguide. An increase in the number of resonance modes complicates the interpretation of the reflection spectrum, especially if the incident angle is different from zero. For a waveguide grating on a glass substrate, whose parameters are indicated above, the thickness should not exceed 2.8 μm. When using a quartz substrate the thickness allowed can be reduced to 1.8 μm.

In this way the approximate method for determining the parameters of the created waveguide grating as a sensitive sensor element for measuring the RI of the analyzed medium was developed. The exact values of the resonant wavelengths at different angles of the incidence were found with the RCWA method. The values are very close to those obtained with the developed approximate method. The theoretical investigations revealed that the sensor sensitivity increases when the angle of incidence increases. It also increases when the RI of the researched medium approaches the average refractive index of the grating.

### 3.2. Fabrication of Photosensitive Nanocomposite Waveguide Layers and Resonant Structures

Theoretical estimates showed that the single-mode waveguides are most suitable for effective functionality of RWS. According to the calculations the thickness of a grating with an average refractive index $n_{g}$ = 1.5255, placed on a substrate with a refractive index $n_{s}$ = 1.5151, should not exceed 2.8 μm.

The solutions of polymers are typically used for the fabrication of thin waveguide layers. We developed new original manufacturing technology for the fabrication of the waveguide resonant structures applying the photocurable monomer blends containing proper amount of the LaPO_4_ NPs (see Section 2.1) without any solvent. In this case, thin light-sensitive layers are formed directly from the low-viscous monomer-NP mixtures. These layers are cured during the subsequent holographic structuring. At the same time, the manufacturing technology should provide controllable and reproducible thickness of the layers and high thickness uniformity. 

Commonly thin polymer layers are prepared by spin-coating of the polymer solution on the substrate with following drying of the layer at constant temperature and humidity. We also used spin-coating to apply the original nanocomposite onto the substrate without using a solvent. The effect of the substrate surface treatment and the spin-coating speed on the thickness and quality of the layer surface was investigated. It was shown that by varying the speed within 2000–3000 rpm, it is possible to produce layers with a thickness of 0.4–1.3 μm. The reproducibility of the layer thicknesses was of about 10%. The chemical cleaning and following O_2_ plasma treatment (0.5–3 min) of the substrate surface provided better adhesion of the monomer bland to the substrate and better layer homogeneity. The disadvantage of this method is that the subsequent holographic structuring of the layer with an open surface must be carried out in an inert gas atmosphere (argon), since atmospheric oxygen blocks the radical polymerization process. This requires the building of a special box for an inert gas within the holographic set up and complicates the technology of the RWS fabrication. The use of the second substrate to protect the liquid layer led to a violation of the surface and uniformity in the thickness. The formation of wedged layers was observed for all tested samples. 

To avoid these drawbacks and to obtain uniform and protected light-sensitive nanocomposite layers, we developed the method in which the formation of the layer takes place between two substrates using the Specac Mini Pellet Press with a controlled load.

**Figure 6:**
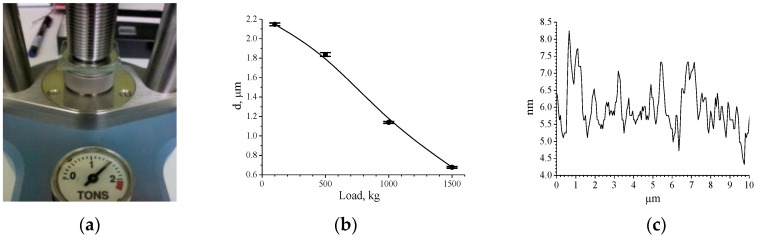
(**a**) A photosensitive layer between glass substrates under the press; (**b**) thickness of the polymer film depending on a load; (**c**) surface profile of the polymer film polymerized between two glass substrates (Y scale 4.5–8 nm), scan: 10 μm × 10 μm.

In this method, a dosed drop of the material is deposited on the glass substrate and then covered with the second one (it is usually pre-treated by an anti-adhesive substance). The layer with the required thickness can be molded using a hydraulic press with a controlled load (Figure 6a). The dependence of the layer thickness on a load is presented in Figure 6b. With increasing load in the range of a 100–1500 kg the thickness of the layers are varied from 2 to 0.7 μm. To achieve the specified dependence, the sample after pressing was polymerized under homogeneous UV radiation, then the treated substrate was removed and the thickness of the layer was measured. 

Thus, the pressing method allows photocurable layers of 0.8–2 μm thickness to be obtained with sufficiently good reproducibility and uniformity of thickness, as well as with a high surface quality. For comparison, we indicate that the roughness of the surface at the polymerization of a layer with an open surface is ±4–5 nm, while the surface roughness of the layers polymerized between two substrates is smaller and amounts to ±1–2 nm (Figure 6c).

The prepared photosensitive layer was immediately exposed to an interference pattern. The holographic recording technique is described in the Section 2.2. As a result of optimization of the exposure conditions, RWS with a thickness of 1.2–1.5 µm and the amplitude of the refractive index modulation $n_{1}$ = 0.01–0.017 were fabricated (Figure 3b). After removing the pretreated substrate, the RWS samples were used for further researches.

**Figure 7:**
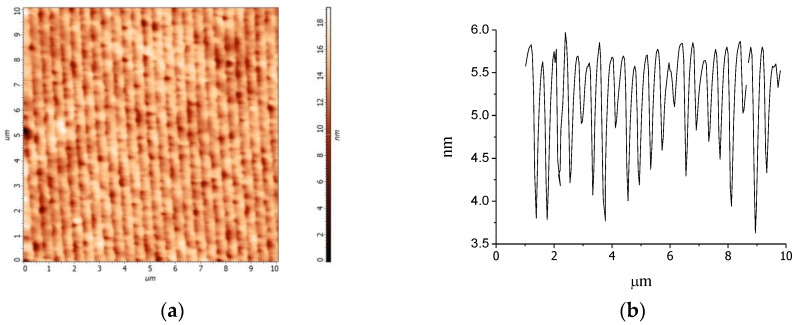
AFM image of the nanocomposite RWS with a thickness 1.45 µm: (**a**) top view; (**b**) surface profile on the distance of 10 µm.

The atomic force microscopy (AFM) images of the grating surface are shown in Figure 7a,b. The AFM measurement exhibits a corrugation, coincided with grating period, and with a height of about 1–4 nm. It is known, that surface corrugation is usually formed on the surface of volume diffraction gratings in the holographic materials possessing a polymerization–diffusion mechanism of the grating recording [46,47]. 

According to our estimations the tiny surface corrugation observed does not influence the coupling of light; and it is not taken into account in the developed theoretical model. 

### 3.3. Resonant Properties of Nanocomposite RWS

The resonant properties of two waveguide grating structures formed on the glass substrates were extensively investigated. The first sample (S1) possessed the following parameters: $n_{s}$ = 1.5151, $n_{g}$ = 1.5255, *d* = 1.3 μm, Λ = 399 nm, $n_{1}$ = 0.017. The second sample (S2) is differed from the first one only in the thickness (*d* = 1.7 μm) and in the value of $n_{1}$ = 0.015. The period of the RWS was chosen in such a way that the resonance peaks were observed in the 500–750 nm spectral range.

When a broadband radiation illuminates the grating, the resonant peaks will be observed in the specular reflection spectrum under the resonant conditions.

The experimental conditions were chosen taking into account the calculated spectral characteristics of the waveguide gratings. The calculated values of the resonance wavelengths for both S1 and S2 samples are shown in Table 2. The calculations were performed at two incidence angles of a plane wave with TE polarization. At normal incidence a single peak (*λ_res_*) with a $\delta \lambda_{0.5}$ should be observed in the specular reflection spectrum. As the angle of incidence increases, two peaks, shifted from the zero resonance towards short, *λ_res_*_1_, and long, *λ_res_*_2_, wavelengths with bandwidths $\delta \lambda_{0.5}^{\prime }$ and $\delta \lambda_{0.5}^{\prime \prime }$, respectively, will be observed in the spectrum. Note that a small expected spectral width of the reflection peaks imposes strict requirements on the resolution of the spectral equipment. The calculations are usually performed for an infinite grating and an infinite plane wave. It is expected that the decrease in the diameter of the light beam and its divergence will lead to the broadening of the resonance peak.

Thus, the measurements were carried out with TE polarized wave at two angles of incidence: close to 0° and at 10°. The beam diameter was equal to 7 mm.

**Figure 8:**
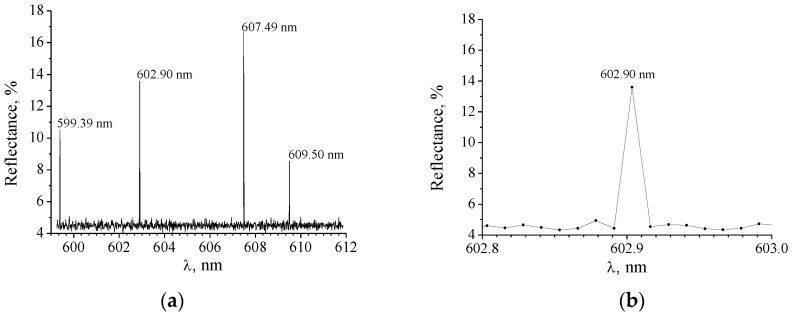
(**a**) Reflection spectrum obtained for the angle of incidence 0.25°; (**b**) the range of the spectrum near the short-wave band.

The reflection spectrum obtained for S1 is shown in Figure 8a,b. The position of the peaks at *λ_res_* = 602.90 nm and 607.49 nm coincides with those predicted theoretically for θ = 0.25°. A part of the spectrum near the short-wave reflection band is also shown in Figure 8b. It is seen that the reflection band corresponds to a single pixel. Thus, $\delta \lambda_{0.5}$ is ≤ 0.012 nm and is limited by the resolution of the spectral equipment. 

The reflection coefficients of the short-wave and the long-wave bands were found as 14% and 17%, respectively. The Fresnel background reflection at the air/grating interface is approximately 4.8%, which corresponds to the calculated data for reflection of a medium with the RI of 1.525. We obtained lower values for the reflection coefficients than those predicted theoretically (of about 98%). The observed decrease in the peak intensity can be the result of both limiting diameter of the light beam and averaging the intensity over the spectral range, corresponding to a single pixel.

It was found that aside from the indicated peaks, two additional peaks were observed, at *λ* = 599.39 nm and 609.50 nm. Their origin is currently unclear. Perhaps they arise from the implementation of the resonance conditions for the waves diffracted to the first and higher orders. However, this issue requires further study.

The wavelength of the resonances observed at θ = 10° (536.55 nm and 674.46 nm) agree well with those calculated theoretically; the spectral widths of both bands are ≤0.012 nm. The corresponding reflection coefficients were 20% and 23%. An increase in the reflection coefficients can be connected with an increase in the real FWHM of the reflection band, which is not resolved with our spectrometer. In addition, a tendency to increase the intensity of the reflection peaks with increasing incident angle was also observed in the waveguide structures based on surface relief gratings.

The same regularities were observed in the reflection spectra measured for sample S2. The values of the resonant wavelengths coincide with those predicted theoretically. The $\delta \lambda_{0.5}$ of the resonance peaks also did not exceed 0.012 nm. The only difference was an increase in the reflection coefficients by 5–7%, which can be explained by increasing the value of $n_{1}d$ and the diffraction efficiency of the grating, respectively.

The quality factor (Q-factor) of the RWSs under study determined for different resonances as Q = $\lambda_{res}/\delta \lambda_{0.5}$ varies in a range of 50,456–56,205. Thus, the waveguide structures fabricated in the organic-inorganic nanocomposite are characterized by Q-factor above 50,000. The theoretical limit for the Q-factor ($\delta \lambda_{0.5}$ = 0.003) lies in the range of 170,000–225,000.

### 3.4. Investigation of the Sensing Properties of RWS 

In order to study the sensing properties of the RI sensor we used sample S1, whose parameters are shown in Table 2. The glass substrates were used for the waveguide gratings, since we developed a surface treatment method to increase the adhesion of the polymer layer to glass. The processing methods for other surfaces, as quartz or an intermediate polymer layer, require further development.

**Figure 9:**
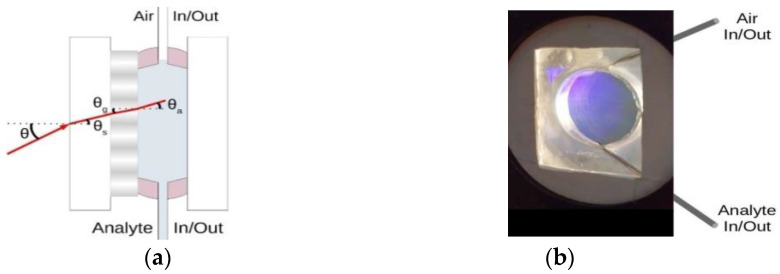
(**a**) Schematic sketch of the fluid cell with the beam propagation; (**b**) the image of the cell. Standard method: θ = constant, θ_a_ varies for different analytes. Modified method: θ_a_ = const, θ varies for different analytes.

A special cell was made for the measurements (Figure 9). The cell consisted of a grating on the substrate, a silicone spacer of 3 mm thickness and a second substrate, limiting the volume of the tested liquid. The cell was placed in the holder with clamps, which ensured its tightness. The cell was filled with liquids using a syringe located in the lower part of the cell. Air was removed from the cell through a second syringe located in the upper part of the cell, ensuring complete filling of the cell with the studied liquid. The complete filling of the cell provided a contact of the entire surface of the grating with the liquid.

The cell was installed into the optical setup described in the Section 2.3. The halogen lamp with maximum intensity at about 670 nm was used as a light source. The specular reflection spectrum from the sample corresponding to the zero order of diffraction of the light wave by the grating was studied. We measured the dependence of the long-wave peak wavelength on the RI of the liquid under study.

To study the sensitivity of the sensor in a wide range of the refractive indices, a set of the liquids of different RI was applied. Their RIs previously measured are shown in Table 3. The maximum allowable value of $n_{a}$ is determined by the average RI of the waveguide-grating. When the RI of the analyte approaches $n_{g}$ = 1.525, the system becomes unstable. The propagation conditions of the waveguide modes are violated and the resonances in such a system cannot be excited. 

The choice of the liquids was determined by the effect of a liquid on the grating. We studied only the liquids which that did not destroy the grating and did not affect its parameters upon contact for four hours.

We investigated two options for the resonance excitation. In the first case the light wave with TE polarization is directed to the cell at an angle θ, which remains constant for all analytes under study (Figure 9a). This geometry is commonly used for the RI sensors. Theoretical estimations of the sensitivity of such a sensor showed that it increases with increasing the angle of incidence. In addition, with a change in the RI, the spectral shift of the long-wavelength resonance peak exceeds the shift of the short-wavelength peak. Therefore, the following measurement conditions were chosen for the experimental studies. We determined the sensitivity of the sensor by measuring the spectral position of the long-wavelength resonant peak at various values of $n_{a}$. The angle of incidence of the light beam in air, θ, was 20°. We did not observe a change in the FWHM of the resonance peak, which was 0.012 nm for different $n_{a}$. The reflection coefficients were varied in the range of 8–10%. There is a decrease in the reflection coefficient for the grating in contact with the analyte compared with the values given above for the grating contacting with air. This may be due to additional reflection and scattering losses from the substrate, which have not been fully taken into account. Note that the value of the reflection coefficient is not critical when measuring $n_{a}$.

**Figure 10:**
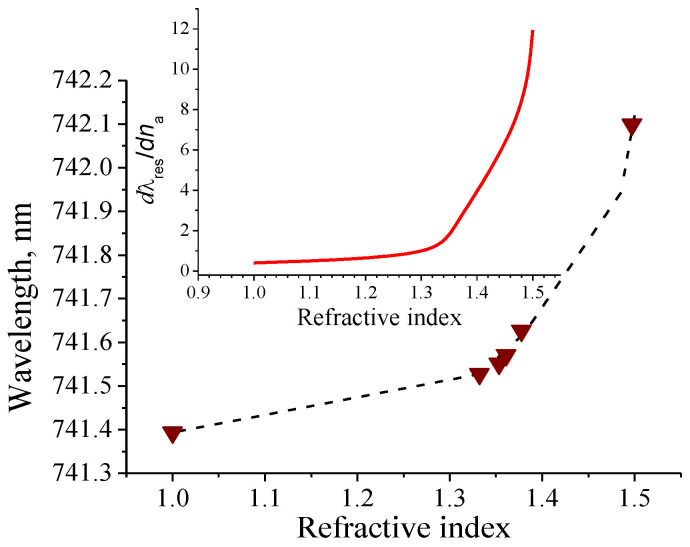
Experimentally measured spectral positions of the resonant peaks (triangular dots) obtained for the incidence angle 20° and dependence of the resonance wavelength on the RI calculated by the rigorous coupled-wave analysis (RCWA, dashed line). The inset shows $d\lambda_{\mathrm{res}}/dn_{a}$.

The results of the measurements are shown in Figure 10. The theoretically calculated dependence of *λ_res_*($n_{a}$) at the incident angle of 20° is also displayed. 

There is a good agreement of the measurement results with the theoretical ones. The *λ_res_*($n_{a}$) dependence is non-linear, so the sensitivity of the sensor changes with changing of $n_{a}$. The inset shows the first derivative, $d\lambda_{res}/dn_{a}$, which characterizes the sensitivity of the sensor. This depends on the RI of the analyte and increases when its value approaches the average RI of the grating. The defined sensor sensitivity in this case varies within 0.4–12 nm/RIU. One-pixel shift in the peak position corresponding to 0.012 nm can be measured with our measurement system. As a result, a minimum detectable change in RI, Δ$n_{min}$, also varies in a range of 0.0279–0.0010 RIU. Accordingly, the range of the FoM change lies in the range of 33–1000 RIU^−1^. The value of $\delta \lambda_{0.5}$ = 0.012 nm was used to calculate FoM. 

**Figure 11:**
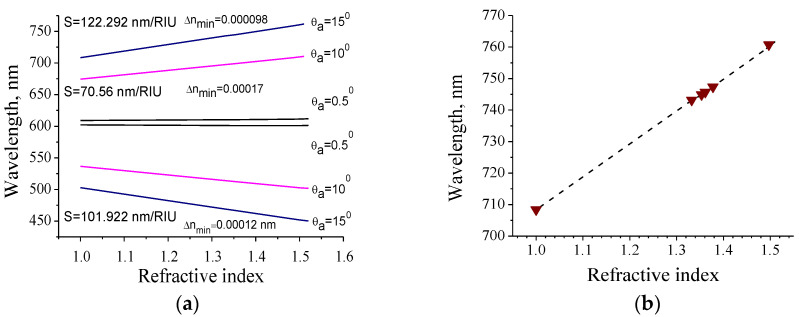
(**a**) Dependence of the resonance wavelength on the RI calculated by the RCWA at different θ_a_. The values of θ_a_, sensor sensitivity S and minimal Δ$n_{min}$ are shown near the corresponding lines; (**b**) The experimentally measured spectral positions of the resonance peaks (triangular dots) obtained at θ_a_ = 15° and dependence of the resonance wavelength on the RI calculated by the RCWA (dashed line).

We also investigated the case where the angle of the beam propagation in a liquid (θ_a_) was kept constant. By changing the angle θ, we were able to determine the resonance wavelength at a constant angle θ_a_. The theoretically calculated dependencies of the resonance wavelength on the refractive index of the medium for different θ_a_ are presented in Figure 11a. In this case the sensor sensitivity also arises with increasing θ_a_. Herewith a long-wave reflection peak is more sensitive to the changes of $n_{a}$.

As can be seen from Figure 11a, the dependence *λ_res_* ($n_{a}$) can be approximated by a straight line. Such a linear dependence allows creating a computer program in order to calculate $n_{a}$ corresponding to the *λ_res_*, and vice versa, with high accuracy. For θ_a_ = 15° and 1 ≤ $n_{a}$ ≤ 1.5, θ varies in the range of 15°–22.844°. This corresponds to the change in the resonance wavelength in the range of 708.34–761.73 nm. Thus, using the law of refraction, we can calculate the external angle of incidence θ for each $n_{a}$ indicated in Table 3. By measuring the reflection spectrum for each angle θ, we have determined the wavelengths of the long-wavelength resonance peaks. The results are shown in Figure 11b. There is a very good agreement between the measured values of *λ_res_* and the theoretically predicted ones that confirms the applicability of the proposed method. For current geometry the estimated values are: S = 122 nm/RIU, FoM ≈ 10,167 RIU^−1^, Δ$n_{min}$ ≈ 1 × 10^−4^ RIU. It is important to note that this limitation is determined by the detection system used. The theoretical limit of the RI change is predicted to be one order of magnitude lower.

The method is convenient for measuring the RI of various solutions with a low concentration of the analyte. The measured resonance wavelength for the solvent with a known RI serves as a starting point for the measurement of the solution’s RI. The accuracy of the result can be verified by the calculation of θ_a_ using the refraction law equation. If at the measured values of θ and $n_{a}$ θ_a_ ≠ 15°, it is necessary to correct θ, *λ_res_* and, accordingly, $n_{a}$. The procedure should be repeated until the value θ_a_ = 15° is obtained.

In this case Δ$n_{min}$ is determined not only by the resolution of the spectral equipment, but also by the accuracy of the incidence angle measurement. The estimations show that Δ$n_{min}$ = 1 × 10^−4^ RIU with the accuracy of the incidence angle measurement of 6″, which is provided by modern goniometers. In our studies, the accuracy of the angle measurement was 20″, while Δ$n_{min}$ is of about 2 × 10^−4^ RIU.

We measured the RI index of the 2 vol.% ethanol-water solution. After several measurements of the angle θ for the solvent and for the solution, the average value of $n_{a}$ = 1.3326 ± 0.0002 was obtained. This was also verified by the measurements with an Abbe refractometer.

## 4. Discussion

The main characteristic of RI sensor is the minimum detectable change in the refractive index, Δ$n_{min}$ (or limit of detection). Δ$n_{min}$ = Δ*λ*/S, where Δ*λ* is the minimum detectable spectral shift of resonances, the value of which depends on the resonant bandwidth and the detection of the resonance maximum. So limit of detection is determined by the sensor design, the RI of the grating and the waveguide, their thicknesses, period and RI modulation amplitude of the grating, as well as by the incident angle and the polarization state of the illuminating wave.

As was mentioned in Introduction, a large number of sensors based on relief structures have been developed and investigated. Here we will indicate several examples of sensors which provide a minimal limit of detection. We will focus only on the simplest devices that used one-dimensional waveguide gratings and the wavelength shift detection schemes. We will not consider sensors based on interferometers, microfluidic systems, Bragg grating devices and others that provide a spectral sensitivity of 10^4^ nm/RIU and limit of detection of 10^−5^–10^−8^ RIU, for example, in the review [34] and links therein.

Back in 1996 using a grating fabricated in thick fused silica disk, Δ$n_{min}$ of the order of 10^−4^ RIU was experimentally achieved; the theoretical limit of detection was 10^−6^ RIU [30]. In [48] the diffraction grating with groove depth of approximately 50 nm was deposited on a dielectric waveguide layer (tantalum pentoxide, Ta_2_O_5_) of 120 nm thickness over the glass plate. It was found that the detection limit of the biosensor for the changes in medium’s RI was approaching 10^−5^ RIU; Δ*λ* was 0.01 nm. Also, a resonance shift of 0.01 nm was recorded in [49] for a resonant sensor fabricated in polymer imprinted with submicron grating patterns (~500 nm grating periods) and coated with a high-index dielectric material (such as TiO_2_ or HfO_2_). It should be noted that for the fabrication of the aforementioned sensors, multistage lithographic and embossing technologies were used.

By combining the analytical model and numerical simulations, in [38,50], the optical response of the sensing device based on grating-waveguide was modeled. In the first case, a simple 1D-all-dielectric nano-slit array on a substrate was considered as a sensitive element for TM polarized light wave. The RI of the grating and substrate were $n_{g}$ = 1.6 and $n_{s}$ = 1.5, respectively. Varying the structural parameters and the incident angle of the light wave it was established that by choosing an optimal grating period, relief depth, filling factor and the incident angle, FoM can be increased up to 12,000 and, respectively, limit of detection can be decreased up to 8 × 10^−5^ RIU.

In the second case, two variants of the structure were studied: a grating and a waveguide on a substrate and only a grating on a substrate. The modeling was performed for a structure made of a high-index material, such as silicon nitride (Si_3_N_4_, $n_{g}$ = $n_{wg}$ = 2.00) immersed in water ($n_{c}$ = 1.333) and with silicon dioxide (SiO_2_, $n_{s}$ = 1.45) as a substrate. The waves with TE and TM polarization at normal incidence on the grating were used. The period and thickness of the grating and the thickness of the waveguide varied. The dependencies of the FWHM of the resonant reflection band, angular and spectral sensitivity, and FoM on the indicated parameters were obtained. It was established that the highest spectral sensitivity and FoM can be achieved using a sensor in which the grating is a waveguide; S*_max_* = 325 nm/RIU, FoM = 1626 RIU^−1^, Δ$n_{min}$ ≈ 6 × 10^−4^. It was also found that the angular sensitivity of such structures is much higher than the spectral sensitivity. The results can be seen as a roadmap for the optimization and manufacture of high-performance, compact broadband RI sensors.

Compared to the sensors described, the nanocomposite waveguide structure under study is characterized by lower sensitivity; however, due to a low resonance bandwidth, the FoM is close to the values indicated above. The theoretical limit of detection is 10^−5^ RIU. The value was not achieved because of the limited spectral resolution of the measurement equipment as it was stated earlier.

The performance of the sensor under study is close to those obtained for photonic crystal cavity RI sensors [35]. The RI sensitivity of the latter lies in the range of 50–400 nm/RIU, FoM varies from several tenths to 10,000 RIU^−1^. Photonic crystal nanobeam cavities are the best performing ones [see references [51,52,53,54] and references therein]. The photonic crystal nanobeam cavity based on a polymer exhibited the RI sensitivity of 386 nm/RIU, the FoM of 9190 RIU^−1^ and Q = 36,000 [53]. The photonic crystal cavity also achieved the record high Q-factor value of 7 × 10^5^ [52]. We note that we achieved the Q-factor exceeding 5 × 10^4^, which is mostly limited by the spectral resolution of the measurement equipment as was specified above.

We would like to emphasize that in this work we did not aim to manufacture a sensor for practical applications. Our main task was to establish the possibility to use holographic nanocomposite RWS for sensing applications. 

The nanocomposite RI sensors have the potential for further improvement of their sensitivity. According to the theoretical estimations, the sensor sensitivity can be significantly increased both by reducing the RI of the substrate and by applying a high RI layer on top of the grating surface, for instance, graphene or TiO_2_ layers. It is also possible to reduce the limit of detection by using TM-polarized light waves for measurements, for which the resonance bandwidth is two orders of magnitude smaller than for TE-polarized waves. Moreover, the sensitivity may be enhanced by increasing the grating refractive index modulation amplitude using inorganic nanoparticles possessing higher RI or noble metal nanoparticles. The improvement of the angular and spectral resolution of the equipment is also necessary to fully realize the capabilities of the current sensors. The stated methods are a subject of future investigation.

## Figures and Tables

**Table 1:** The resonant wavelengths and sensitivities calculated for several refractive indexes (RI) of the analyzed medium.

$n_{a}$	λ_res_, nm	S_n_, μm^−1^	S_λ_, μm^−2^	S, nm/RIU	FoM, RIU^−1^	*S*_θ_, deg/RIU
1.000	804.0984	0.0157	–14.791	0.80	267	0.133
1.332	804.2528	0.0186	–14.798	0.95	317	0.158
1.350	804.2730	0.0216	–14.798	1.10	367	0.183
1.400	804.3505	0.0361	–14.795	1.80	600	0.306
1.450	804.4959	0.0778	–14.791	3.96	1320	0.659
1.500	804.9512	0.3973	–14.775	20.20	6733	3.368
* 1.500	802.7418	1.2490	–14.900	63.90	21300	10.090

* The substrate was covered with a buffer layer with the *n* = 1.380 and thickness of 1 μm.

**Table 2:** The resonant characteristics of the RWS structures.

Sample	θ = 0°, TE		θ = 10°, TE			
	λ_res_, nm	$\delta \lambda_{0.5}$, nm	λ_res1_, nm	$\delta \lambda_{0.5}^{\prime }$, nm	λ_res2_, nm	$\delta \lambda_{0.5}^{\prime \prime }$, nm
S1	605.342	0.003	536.559	0.002	674.459	0.002
S2	606.300	0.002	537.330	0.0009	675.308	0.001

**Table 3:** The analytes under study and their RI.

Analyte	Refractive Index, *n_a_*
Water	1.3321
60 vol.% ethanol-water mixture	1.3532
Ethanol	1.3611
Isopropanol	1.3776
Toluene	1.4970
